# Supplementary material for: Vitamin D Modified DSS-Induced Colitis in Mice via STING Signaling Pathway
Source: Biology (Basel). 2025 Jun 18;14(6):715. doi: 10.3390/biology14060715 (PMC12190092; doi:10.3390/biology14060715)
Supplement: Supplementary file 1 [file biology-14-00715-s001.zip › Supplementary Materials S1: 16s-report of gut microbiota/Groups/Lefse_Analysis/biomarkers_raw_images/1_d__Bacteria-p__Firmicutes-c__Clostridia-o__Oscillospirales-f__Oscillospiraceae-g__Flavonifractor.pdf]

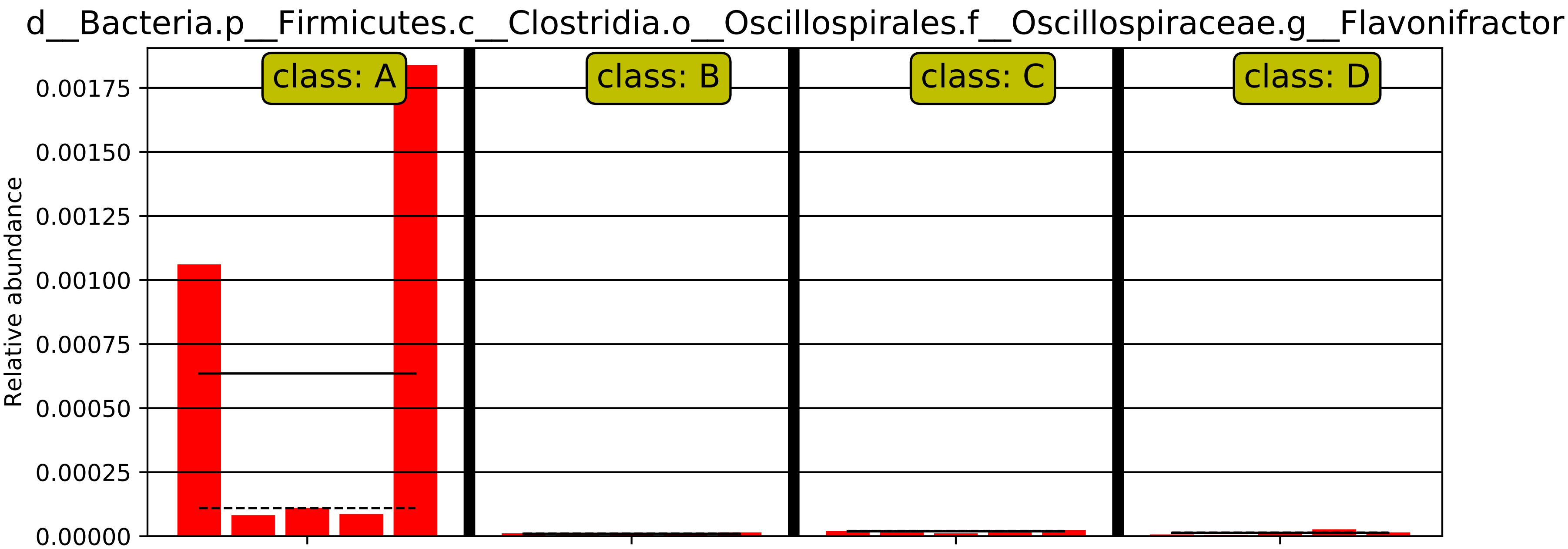
d__Bacteria.p__Firmicutes.c__Clostridia.o__Oscillospirales.f__Oscillospiraceae.g__Flavonifractor
class: A
class: B
class: C
class: D
Relative abundance
0.00175
0.00150
0.00125
0.00100
0.00075
0.00050
0.00025
0.00000
